# Multi-Objective Optimization and Mechanical Properties Analysis of Steel–PVA Hybrid Fiber-Reinforced Cementitious Composites

**DOI:** 10.3390/ma17174324

**Published:** 2024-08-31

**Authors:** Rui Wang, Pinle Zhang

**Affiliations:** Faculty of Civil Engineering and Architecture, Kunming University of Science and Technology, Kunming 650599, China; 20110165@kust.edu.cn

**Keywords:** principal component analysis, factor optimization method, steel fiber, PVA fiber, multi-objective optimization, mechanical property analysis

## Abstract

When steel fiber and PVA fiber produced in China and PVA fiber made in Japan are prepared according to the appropriate proportions, the mechanical properties of hybrid fiber-reinforced cementitious composites (HFRCC) are better, which is beneficial to cost control and has wide application prospects. The effects of the volume content of steel fibers and the volume substitution rate of PVA fibers on the tensile strength, compressive strength, and flexural strength of HFRCC were analyzed using the factor optimization method and principal component analysis (PCA). Through the principal component analysis of HFRCC, a mathematical model for comprehensive performance evaluation was established, and a multi-objective optimization was carried out. The results show that compared with the matrix, the tensile strength, compressive strength, and flexural strength of concrete increase significantly when the volume content of steel fibers is 0.2–0.4% and the volume substitution rate of domestically produced PVA fibers in China or PVA fibers produced in Japan is 50–100%. The maximum cost reduction is 88.25%, and the strength index of HFRCC can reach the optimum; the weights of each factor on the performance of HFRCC were obtained through mathematical statistics. Combined with a variable correlation analysis, these indicators should be noted when optimizing the performance of HFRCC. The research results can provide a basis for the preparation of HFRCC.

## 1. Introduction

In recent years, with the vigorous development of the world’s infrastructure, requirements for the main building materials are getting higher and higher. Meanwhile, there is a high demand on the construction industry to increase the sustainability of their materials and methods. This development has led to the development of new smart materials with superior mechanical properties, durability, and sustainability [1,2,3]. Hybrid fiber-reinforced cementitious composites (HFRCC) are those that incorporate two or more randomly distributed fibers into the cement mortar. Thus, a new type of engineering cement-based composite material with good tensile, bending, cracking, shrinkage and impact fatigue properties can meet the requirements of the high safety and high durability of materials for the rapid development of infrastructure in various countries [4,5,6,7]. The relevant research shows that steel fiber can significantly improve the tensile properties and compressive strength of cement-based materials, and greatly improve the durability of materials. Steel fiber concrete has the advantages of high impact and seismic resistance [1,4,8,9,10,11,12,13,14,15,16]. Polyvinyl alcohol (PVA) fiber has the characteristics of high strength, a high elastic modulus, acid and alkali corrosion resistance, environmental friendliness, etc. It has the effect of strengthening, toughening, and improving the cracking resistance of cement-based materials [17,18,19,20,21,22,23,24].

At present, most of the PVA fibers on the market are produced by the Japanese Kuraray company; the cost per cubic meter of cement-based materials prepared with it is about 10 times that of ordinary concrete [25,26], so it is very difficult to use on a large scale in actual projects, and the unit cost of China’s production of PVA fibers is low, and the cost per cubic meter of cement-based materials prepared with it is about 2 times that of ordinary concrete [27,28]. Current studies mainly consider single-doped steel fiber, single-doped PVA fiber, or mixed steel fiber and PVA fiber. In this study, steel fiber, PVA fiber produced in China, and Kuraray PVA fiber made in Japan were prepared according to scientific proportions, and a mix ratio optimization analysis was carried out to improve the uniformity of fiber spatial distribution and make fiber properties complement each other, so as to improve the fiber reinforcement efficiency and give full play to the hybrid effect between fibers [29,30,31,32,33,34]. While making up for the defects of a single fiber, it can greatly reduce the cost, solve the problems existing in ordinary cement-based materials, and make it possible to apply it in large-scale projects [35,36,37,38,39,40].

Existing studies on HFRCC mainly focus on measuring material performance indicators with a single performance strength index, but the reality is that multiple material performance indicators usually need to be evaluated in actual engineering, and the material performance indicators are often in conflict with each other and have mutual constraints, which requires a comprehensive evaluation of material performance indicators. Principal component analysis (PCA) is a statistical method that converts multiple variables into several uncorrelated comprehensive index variables by dimensionally reducing high-dimensional variables and using multivariate statistical analysis as the medium. These transformed comprehensive index variables are the main components of many complex variables [41,42]. There are two main purposes: One is to explain the correlation of the original variable by finite potential variables. Second, variable classification and mathematical statistical processing are carried out to express most of the information with a limited number of indicators [43,44].

According to the existing research results, the fiber volume content has a great influence on the properties of ordinary cement-based materials [21,45,46,47,48,49,50,51,52,53]. In this study, the factor optimization and principal component analysis methods are combined to carry out a comparative optimization analysis and multi-objective optimization of HFRCC. The internal relationship between HFRCC steel fiber volume content, PVA fiber volume content, tensile strength, compressive strength, and bending strength was determined, and the optimal volume replacement rate of PVA fiber produced in China to PVA fiber produced in Japan was obtained, so as to reduce the preparation cost of HFRCC and combine it with engineering performance, in order to provide a reference for the comprehensive utilization of HFRCC.

## 2. Experiment

### 2.1. Raw Material

**Figure 1:**
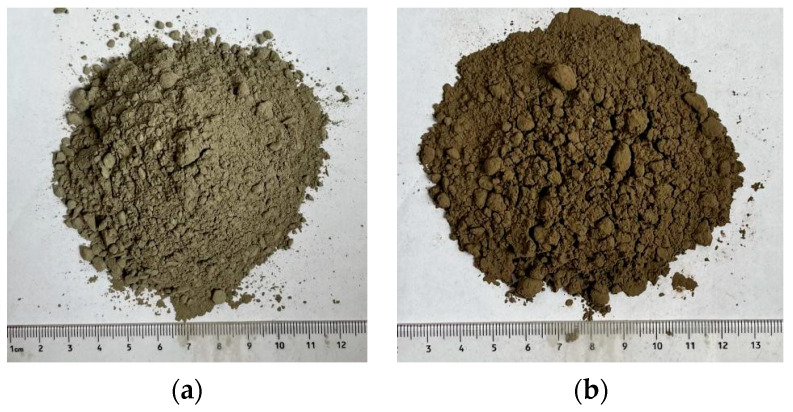
Test material: (**a**) Portland cement; (**b**) fly ash.

**Figure 2:**
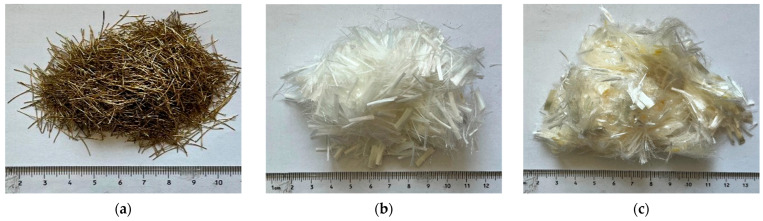
Fiber sample for test: (**a**) Steel fiber; (**b**) Japan PVA fiber; (**c**) China PVA fiber.

The cement is P·O42.5 ordinary Portland cement produced by the Yunnan Xinhua Cement Factory (as shown in Figure 1a) with a density of 3.1 g/cm^3^. Its physical and mechanical properties and chemical composition are shown in Table 1 and Table 2. Fly ash was I grade fly ash produced in Xingyi Power Plant, Guizhou Province, with an apparent density of 2.5 g/cm^3^ and a combustion loss of 3.8%. The sand is quartz sand with a fineness modulus of 2.9, average particle size of 70–110 mesh (140–210 μm) and density of 2.66 g/cm^3^. The fly ash sample is shown in Figure 1b, and its physical properties and chemical composition are shown in Table 3 and Table 4. Polycarboxylic acid superplasticizer produced by Hongxiang building admixture factory was used as superplasticizer. The steel fiber is made of smooth round flat copper-plated microwire steel fiber produced by Hebei Hengshui Maole Metal Products Co., Ltd. (Hengshui, China). The performance indexes are shown in Table 5. The imported PVA fiber is K-I PVA fiber (hereafter referred to as Japan PVA fiber) produced by the Kuraray Company in Japan, and the performance indicators are shown in Table 5. The domestic PVA fiber produced in China is produced by Shanghai Yingjia Company (hereafter referred to as China PVA fiber, Shanghai, China), and the performance indicators are shown in Table 5. The water is Kunming local tap water. The test fiber sample is shown in Figure 2.

### 2.2. Performance Measurement

#### 2.2.1. Dumbbell Axial Tensile Test Method

**Figure 3:**
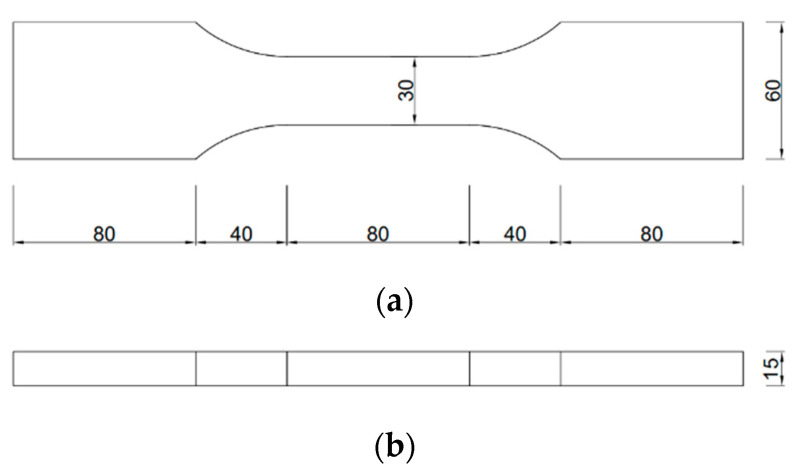
Dumbbell axial tensile specimen size: (size unit in the figure: mm) (**a**) Plane graph; (**b**) side view.

**Figure 4:**
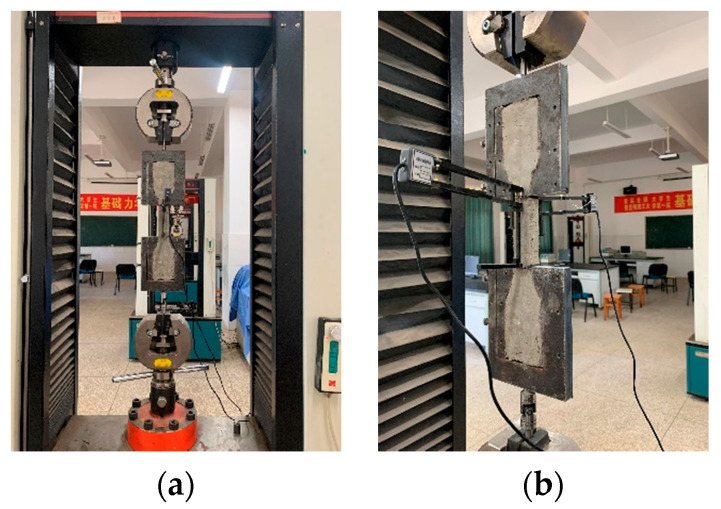
Dumbbell type axial tensile test device: (**a**) Front view; (**b**) side view.

The tensile strength test adopts the relevant provisions of JG/T2461-2018, “Test Method for Mechanical Properties of High ductility Fiber-reinforced cement-based Composite Materials” [54], and sets 3 parallel specimens for each mix ratio, dumbbell type specimens with a middle tensile section size of 30 mm × 15 mm and effective tensile standard distance of 90 mm, as shown in Figure 3. The specimen was continuously and uniformly loaded at a speed of 0.15 mm/min, and the test device is shown in Figure 4.

#### 2.2.2. Cube Compression Test Method

**Figure 5:**
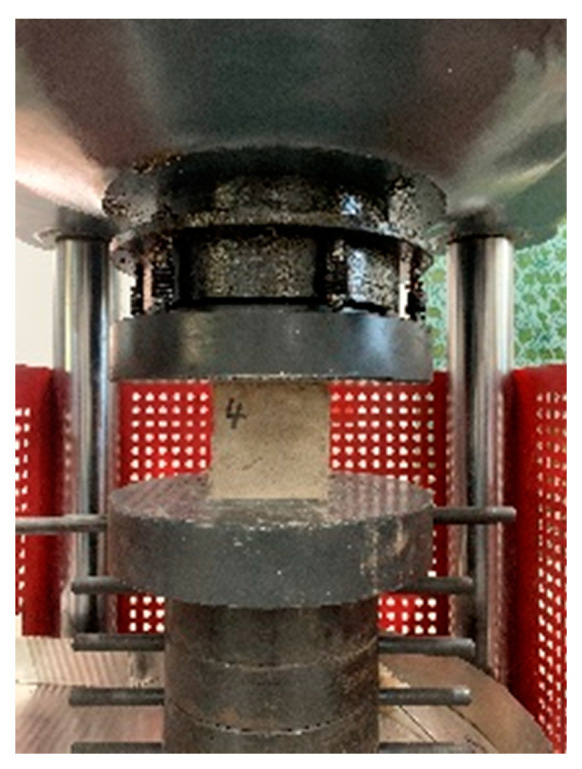
Cube compression test device.

The compressive strength test adopts the relevant provisions of JG/T2461-2018 “Test Method for Mechanical Properties of High ductility Fiber-reinforced cement-based Composite Materials” [54]. Three parallel specimens are set for each mix ratio, and the specimen size is 100 mm × 100 mm × 100 mm cube specimen. The specimen was continuously and uniformly loaded at a speed of 0.15 mm/min, and the test device is shown in Figure 5.

#### 2.2.3. Four-Point Bending Test Method for Sheet Metal

**Figure 6:**
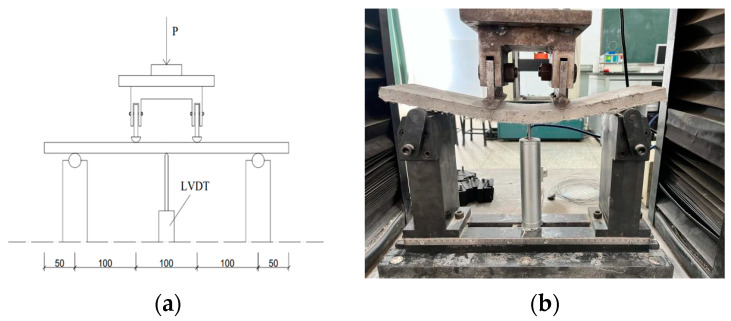
Four-point bending test diagram for sheet metal: (**a**) Installation diagram; (**b**) flexural test diagram.

The flexural strength test adopts the relevant provisions of JG/T2461-2018 “Test Method for Mechanical Properties of High ductility Fiber-reinforced cement-based Composite Materials” [54]. Three parallel specimens are set for each mix ratio, and the specimen size is 400 mm × 100 mm × 15 mm flat specimen. The specimens are continuously and uniformly loaded at a speed of 0.5 mm/min. The test device is shown in Figure 6.

## 3. Results and Discussion

In this experiment, the factor optimization method was used to design the test mix. Under the condition of determining the matrix strength, the three factors of steel fiber volume content, PVA fiber volume content produced in China, and PVA fiber volume content produced in Japan per day were taken as variables in the HFRCC mix design. The influence of the volume content of PVA fiber produced in China and the volume content of PVA fiber produced in Japan on the mechanical properties of HFRCC was studied by the single factor optimization method, and the optimal content of PVA fiber in HFRCC was determined. On this basis, through the multi-factor optimization method, the appropriate volume content of steel fiber and the optimal volume content of PVA were selected, and the fiber hybrid test was conducted on the volume content of PVA fiber produced in China with a replacement rate of 0%, 25%, 50%, 75%, and 100% of PVA fiber produced in Japan. The steel fiber, PVA fiber produced in China, and PVA fiber produced in Japan were prepared according to scientific proportions, and the optimal mix ratio was analyzed. The matrix compressive strength for HFRCC is not less than 30 MPa, the matrix mix ratio of predetermined HFRCC is cement fly ash/sand/water = 1:1.8:0.6:0.56. Due to the difficulty of the test and the high degree of dispersion, to ensure the reliability of the test results, the calculated values of the ultimate tensile strength and compressive strength need to be multiplied by the conversion factor 0.95. The calculated bending strength should be multiplied by the conversion factor 0.85. The average strength of the three specimens was taken as the final result.

### 3.1. Single Factor Optimization Method PVA Fiber Volume Content Test Design and Strength Analysis

**Figure 7:**
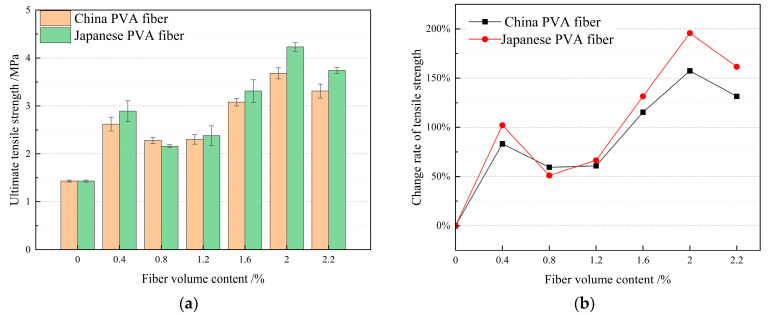
Analysis and statistics of tensile strength of HFRCC with different PVA fiber volumes: (**a**) Tensile strength comparison; (**b**) comparison of changing rates of tensile strength.

**Figure 8:**
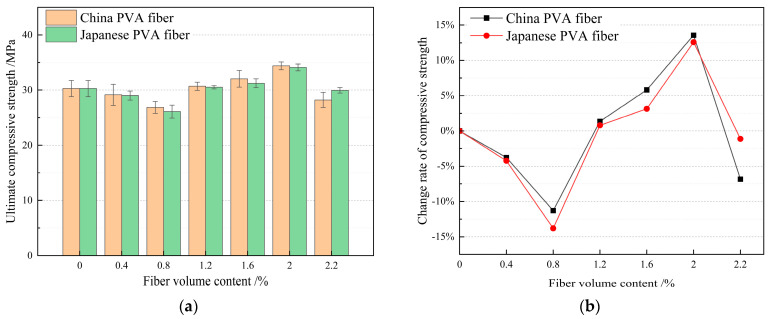
Analysis and statistics of compressive strength of HFRCC with different PVA fiber volumes: (**a**) Compressive strength comparison; (**b**) comparison of compressive strength change rates.

**Figure 9:**
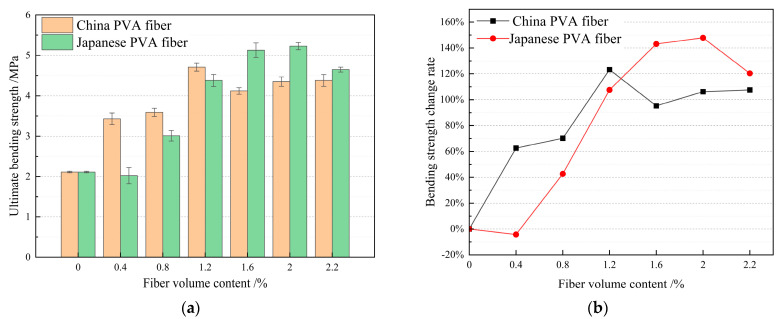
Analysis and statistics of bending strength of HFRCC with different PVA fiber volumes: (**a**) Comparison of flexural strength; (**b**) comparison of bending strength change rates.

Combined with the previous research results and existing research results [55,56,57,58,59], the volume content of PVA fiber produced in China and PVA fiber produced in Japan is considered to range from 0.2% to 2.2%, and the single factor optimization method PVA fiber volume content test design is shown in Table 6. According to the above sample preparation and performance testing methods, the tensile strength, compressive strength, and bending strength values under different mix ratios were determined and calculated. The test results are shown in Table 6. According to the test results in Table 6, histogram statistics of HFRCC tensile strength, compressive strength, and bending strength for PVA fiber volume content from different sources (produced in China and Japan) are drawn. At the same time, the HFRCC mechanical properties for PVA fibers from different sources (produced in China and Japan) with the same volume content were compared, the performance gap was calculated, and a line chart was drawn, as shown in Figure 7, Figure 8 and Figure 9.

#### 3.1.1. Effect of PVA Fiber Volume Content on Tensile Strength of HFRCC

The histogram of Figure 7a shows that the tensile properties of HFRCC change with the volume and content of PVA fiber. When the volume content of PVA fiber produced in China and Japan is 0.4% and 2.0%, respectively, the tensile strength of HFRCC reaches different peaks, indicating that the design of the volume content range of PVA fiber meets the requirements, and this range includes the optimal volume content of PVA fiber. Compared with the tensile strength of the matrix, the tensile strength of all test groups has been improved to a certain extent, indicating that the addition of PVA fiber can form a three-dimensional network structure, effectively resist the crack expansion of HFRCC, improve the ductility and toughness of HFRCC, and increase its overall strength. It should be noted that within a certain range, the right amount of PVA fiber volume can improve the tensile strength of concrete. However, too high or too low a fiber content may lead to irregular changes in tensile strength. High fiber content may lead to interference between fibers and affect the effective function of the fibers. However, too low a fiber content may not form enough fiber network structure to effectively improve the tensile strength. Based on the same PVA fiber volume content, the influence of PVA fiber volume content produced in China and Japan on the tensile strength of HFRCC was analyzed. In the case of the same PVA fiber volume content, compared with PVA fiber produced in China, PVA fiber produced in Japan has a more significant impact on the tensile properties of HFRCC. When the volume content of PVA fiber is 2.0% and 2.2%, the increase effect of PVA fiber produced in Japan on the tensile strength of HFRCC is 13.03% and 11.68%, respectively, compared with that of PVA fiber produced in China. The increase effect of PVA fiber produced in Japan on the tensile strength of HFRCC is more significant. It should be noted that when the volume content of PVA fiber is 0.8%, PVA fiber produced in China has a better effect on the tensile strength of HFRCC than PVA fiber produced in Japan, but there is little difference in the tensile strength between the two, and the tensile strength difference is only 0.12 MPa.

The tensile strength of HFRCC and matrix (blank group C0) concrete with the same volume of PVA fiber from different sources (produced in China and Japan) is compared and analyzed, as shown in the line chart in Figure 7b. When the volume content of PVA fiber is 0–1.2%, the change in tensile strength of HFRCC shows a nonlinear trend. The analysis shows that the dispersion of single-doped PVA fiber in the concrete matrix may not be ideal, resulting in an uneven distribution of fiber in concrete, with high fiber content in some areas and low fiber content in other areas. This results in irregular changes in tensile strength. At the same time, the tensile strength of HFRCC is more sensitive to the influence of external adverse factors, such as fiber dispersion, curing conditions, preparation technology, etc. Therefore, only the PVA fiber content range of 1.2–2.2% was analyzed.

When the volume content of PVA fiber is 2%, the HFRCC tensile strength of PVA fiber produced in China and PVA fiber produced in Japan reaches the maximum value, which is 3.68 MPa and 4.23 MPa, respectively. Compared with the matrix tensile strength, the HFRCC tensile strength is significantly increased by 157.34% and 195.80%, respectively. When the volume content of PVA fiber is 2.2%, the HFRCC tensile strength of PVA fiber produced in China and PVA fiber produced in Japan reaches a large value, which is 3.31 MPa and 3.74 MPa, respectively. Compared with the matrix tensile strength, the HFRCC tensile strength is significantly increased by 131.47% and 161.57%, respectively.

All in all, the incorporation of PVA fiber can significantly improve the tensile properties of HFRCC, and the impact of PVA fiber produced in Japan on the tensile properties of HFRCC is more obvious. When the volume content of PVA fiber ranges from 2.0% to 2.2%, HFRCC has relatively a high tensile strength, and the tensile strength is greater than 3.30 MPa. The order of tensile strength is as follows: C0J2.0 > C0J2.2 > C2.0J0 > C2.2J0. Within this range, the HFRCC tensile strength reduction of PVA fiber produced in China is 0.37 MPa, the HFRCC tensile strength reduction of PVA fiber produced in Japan is 0.49 MPa, and the optimal PVA fiber volume content is 2.0%.

#### 3.1.2. Effect of PVA Fiber Volume Content on Compressive Strength of HFRCC

According to the histogram of Figure 8a, the compression performance of HFRCC changes with the volume and content of PVA fiber, but the change is not obvious, which may be due to the “cluster effect” between fibers, indicating that the aggregation of fibers forms clumps, resulting in cavities or an uneven distribution in HFRCC, and the “cluster effect” will reduce the effective dispersion of fibers [60,61,62,63,64]. Weakened fiber reinforcement thus limits the improvement of compressive strength. When the volume content of PVA fiber produced in China and Japan is 2.0%, the compressive strength reaches the peak or maximum value, which is improved to a certain extent compared with the compressive strength of the matrix (blank group C0), indicating that the design of the volume content range of PVA fiber meets the requirements, and this range includes the optimal volume content of PVA fiber. According to the analysis of the influence of PVA fiber produced in China and PVA fiber produced in Japan on the compressive strength of HFRCC, compared with PVA fiber produced in Japan, PVA fiber produced in China has a more obvious enhancement effect on the compressive strength of HFRCC with the same PVA fiber volume content. When the volume content of PVA fiber is 2.0%, the HFRCC compressive strength of PVA fiber produced in China and PVA fiber produced in Japan reaches the peak values, which are 34.39 MPa and 34.09 MPa, respectively. Compared with PVA fiber produced in Japan, PVA fiber produced in China has a better effect on improving the compressive strength of HFRCC.

A comparative analysis of the compressive strength of HFRCC and matrix (blank group C0) concrete with the same volume of PVA fiber from different sources (produced in China and Japan) is shown in the line chart in Figure 8b. When the volume content of PVA fiber is 0.4%, 0.8%, or 2.2%, the compressive strength of HFRCC is lower than that of the matrix. The reason is that the “bunching effect” between the fibers leads to the formation of loose fiber clumps inside HFRCC [65,66,67]. These clumps may cause stress concentration, reducing the overall compressive strength of the material. At the same time, the orientation of the fiber in the material will also affect the compressive strength. The orientation of the fiber determines the stress transfer path when it is subjected to external forces. If the orientation of the fiber is inconsistent or the orientation difference between the fibers is large, the stress transfer path may be discontinuous, making the HFRCC form a weak point when it is under pressure. When the volume content of PVA fiber is 1.2%, the compressive strength of HFRCC is not significantly changed compared with that of the matrix, so the only range of PVA fiber volume content is 1.6–2%.

When the volume content of PVA fiber is 1.6%, the HFRCC compressive strength of PVA fiber produced in China and PVA fiber produced in Japan reaches a large value, which is 32.04 MPa and 31.23 MPa, respectively. Compared with the matrix compressive strength, the HFRCC compressive strength is increased by 5.81% and 3.14%, respectively. When the volume content of PVA fiber is 2.0%, the HFRCC compressive strength of PVA fiber produced in China and PVA fiber produced in Japan reaches the maximum value, which is 34.39 MPa and 34.09 MPa, respectively. Compared with the matrix compressive strength, the HFRCC compressive strength is greatly increased, which is 13.58% and 12.58%, respectively.

All in all, PVA fiber incorporation has a “positive effect” and “negative effect” on compressive strength. Moreover, the influence of PVA fiber produced in China on the compressive properties of HFRCC is more obvious. When the volume content of PVA fiber ranges from 1.6 to 2.0%, HFRCC has a high compressive strength, and the compressive strength is greater than 31 MPa, and its order is as follows: C2.0J0 > C0J2.0 > C1.6J0 > C0J1.6. Within this range, the HFRCC compressive strength for PVA fiber produced in China increased by 2.35 MPa. The HFRCC compressive strength for PVA fiber produced in Japan increased by 2.86 MPa, and the optimal PVA fiber volume content was 2.0%.

#### 3.1.3. Effect of PVA Fiber Volume Content on HFRCC Flexural Strength

According to the histogram of Figure 9a, the bending strength of HFRCC changes with the volume content of PVA fiber. When the volume content of PVA fiber produced in China and Japan is 2.0%, the bending strength reaches a peak, indicating that the design of the volume content range of PVA fiber meets the requirements, and this range includes the optimal volume content of PVA fiber. Compared with the matrix, the bending strength is improved to a certain extent, because PVA fiber can effectively prevent the expansion of microcracks in the material. Cracks will form inside the material, and the addition of PVA fiber can form a “bridge” structure around the crack, hinder the expansion of cracks, and help disperse and absorb stress, so as to improve the toughness of the concrete, reduce the generation and expansion of cracks, and improve the crack resistance and bending strength of the material [68,69,70,71].

The bending strength of HFRCC and matrix concrete with the same volume of PVA fiber from different sources (produced in China and Japan) is compared and analyzed, as shown in the line chart in Figure 9b. When the volume content of PVA fiber produced in Japan is 0.4%, the bending strength of HFRCC is lower than that of the matrix, so it is not considered. When the volume content of PVA fiber is 1.6–2.2%, the bending strength improvement effect of HFRCC is more obvious and regular than that of the matrix. Therefore, the only range of PVA fiber content is 1.6–2.2%.

When the content of PVA fiber is 0–1.2%, PVA fiber produced in China has a more significant improvement effect on the bending strength of HFRCC than PVA fiber produced in Japan. When the content of PVA fiber is 1.6–2.2%, the effect of PVA fiber produced in Japan on the bending strength of HFRCC is more significant than that of PVA fiber produced in China. When the volume content of PVA fiber produced in Japan is 2.0%, the bending strength of HFRCC reaches a peak value of 5.23 MPa, and the bending strength of HFRCC increases by 147.87% compared with that of the matrix. When the volume content of PVA fiber produced in China is 2.2%, the bending strength of HFRCC reaches a peak value of 4.38 MPa, and the bending strength of HFRCC increases by 107.58% compared with that of the matrix.

All in all, the incorporation of PVA fiber can significantly improve the flexural performance of HFRCC. When the bending strength is the first objective, the content of PVA fiber produced in China ranges from 2.0% to 2.2%, and the content of PVA fiber produced in Japan ranges from 1.6% to 2.0%. In these cases, HFRCC has a high bending strength, greater than 4.35 MPa, and the order is as follows: C0J2.0 > C0J1.6 > C2.2J0 > C2.0J0. Within this range, the HFRCC bending strength increase of PVA fiber produced in China is 0.03 MPa, the HFRCC bending strength increase of PVA fiber produced in Japan is 0.1 MPa, and the optimal PVA fiber volume content is 2.0%.

In summary, when the volume content of PVA fiber is 2.0%, the tensile strength and bending strength of concrete are the best. Compared with the PVA fiber produced in China, the tensile strength of concrete produced in Japan has an obvious improvement effect on HFRCC. On the contrary, the compressive strength is slightly decreased, and the amplitude is not obvious. When the volume content of PVA fiber produced in China is 2.0% and 2.2%, and the volume content of PVA fiber produced in Japan is 1.6% and 2.0%, HFRCC has a better bending strength. Comparing the two, the bending strength increases by 0.03 MPa and 0.1 MPa, respectively, and the amplitude is not obvious. Based on the three mechanical properties, HFRCC has the best mechanical properties when the volume content of PVA fiber is 2.0%.

### 3.2. Multi-Factor Optimization Method Steel /PVA Fiber Volume Content Test Design and Strength Analysis

According to the single factor test results of PVA fiber volume content, the mechanical properties of HFRCC are the best when the PVA fiber volume content is 2.0%. According to the preliminary research results of the project and the statistics of relevant literature samples [72,73,74,75], the volume content of steel fiber is scheduled to be 0.2% and 0.4%, and the PVA fiber produced in China will be blended with the volume content of PVA fiber produced in Japan with the replacement rate of 0%, 25%, 50%, 75%, and 100%. The values of tensile strength, compressive strength, and bending strength under different mix ratios were determined. The test mix ratio and results are shown in Table 7. According to the test results in Table 7, the histogram statistics of HFRCC tensile strength, compressive strength, and bending strength for PVA fiber produced in China versus PVA fiber produced in Japan under a different volume content of steel fiber were drawn, where C0 was the matrix strength value.

#### 3.2.1. Effect of the Replacement Rate of PVA Fibers Produced in China on the Tensile Strength of HFRCC

**Figure 10:**
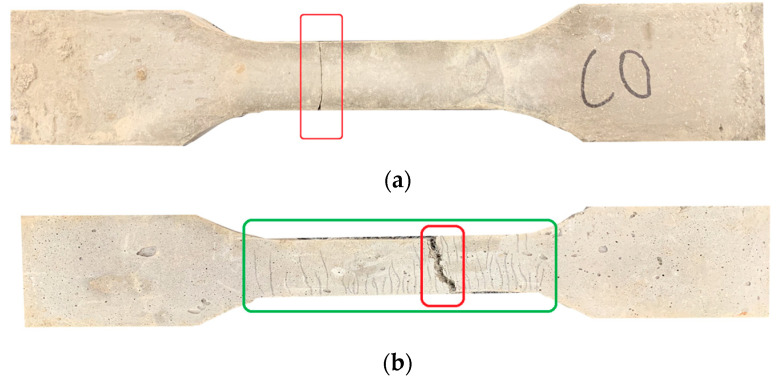
Axial tension crack phenomenon: (**a**) Uniaxial tensile brittle failure mode; (**b**) uniaxial tensile ductile failure mode.

By observing Figure 10, it can be seen that the tensile failure of ordinary concrete and HFRCC is very different. From the beginning of the tensile test to the failure of the specimen, according to the different volume content of fiber, the test failure phenomena are mainly divided into two types: When the volume content of fiber is 0 or low, the failure characteristics of the specimen are brittle failure, or plastic deformation is not obvious, as shown in Figure 10a. After the beginning of the axial tension test, the surface of the test piece does not change significantly, and the tensile load of the test piece shows a linear upward trend. Once the tensile limit stress is reached, the specimen breaks immediately, showing the characteristics of single-slit brittle failure (as shown in the red box in Figure 10a), and the crack propagation is relatively smooth, and the fiber distribution at the fracture surface is uneven or lower. When the fiber volume content is appropriate, the failure characteristics of the specimen are plastic failure, as shown in Figure 10b. In the figure, the green frame is the multi-crack crack zone, and the red frame is the final fracture zone. With the gradual increase in load, the first crack appeared on the surface of the specimen, the increase rate of tensile load of the specimen showed a downward trend, the displacement of the specimen showed an upward trend along the loading direction, and the number of cracks and the width of cracks gradually increased. After reaching the ultimate stress, although the HFRCC matrix was cracked, it was still closely connected with the fibers and had a certain bearing capacity. When the specimen is pulled apart, several parallel cracks appear on the surface of the specimen, showing the characteristics of multi-slit plastic failure, and the integrity of the specimen is good.

**Figure 11:**
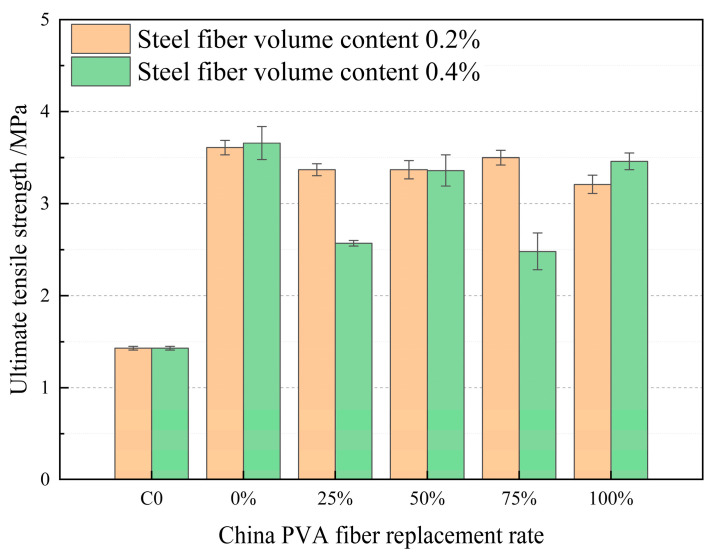
Comparison of tensile strength of PVA fibers produced in China at different volume addition rates.

The analysis of Figure 11 shows that when the volume content of steel fiber is 0.2% and 0.4%, the total volume content of PVA fiber is 2.0%. When the PVA fiber produced in China is used to replace the PVA fiber produced in Japan in HFRCC, the tensile strength of HFRCC is greatly improved compared to the tensile strength of the matrix, which is due to the multi-scale reinforcement effect on HFRCC of mixed steel fiber and PVA fiber (produced in China and Japan). Steel fibers typically have a larger cross-section size and are better suited to resist larger crack propagation, while PVA fibers have a smaller size and are better suited to resist microscopic cracks and improve toughness at the microscale. When cracks occur in HFRCC, the fibers can form a “bridge” structure on both sides of the crack, effectively transferring stress and inhibiting the further expansion of the crack [68,69,70,71]. Steel and PVA fibers can not only bridge smaller cracks, but also provide continuous tensile strength during crack expansion of different sizes, giving HFRCC better tensile strength at different scales. At the same time, blending steel fiber and PVA fiber can produce a synergistic effect. Steel fibers provide a high tensile strength and rigidity, while PVA fibers provide high toughness and good bonding properties. When used in combination, the overall strength and toughness performance can be optimized. When the volume content of steel fiber is 0.2% and the replacement rate of PVA fiber produced in China is 0, that is, the volume content of PVA fiber produced in Japan is 2%, the tensile strength is 3.61 MPa, which is significantly increased compared with the tensile strength of the matrix by 152.45%. When the volume content of steel fiber is 0.4% and the replacement rate of PVA fiber produced in China is 0, that is, the volume content of PVA fiber produced in Japan is 2%, the peak tensile strength is 3.66 MPa, which significantly increases compared with the tensile strength of the matrix by 155.94%. When the volume content of steel fiber is 0.2%, the replacement rate of PVA fiber produced in China is 75%, and the tensile strength of HFRCC is 3.50 MPa, which increases 144.76% compared with the tensile strength of matrix. When the volume content of steel fiber is 0.4%, the replacement rate of PVA fiber produced in China is 100%, and the tensile strength of HFRCC is 3.46 MPa, which increases 141.96% compared with the tensile strength of the matrix. Compared with the steel fiber volume content of 0.4%, when the replacement rate of PVA fiber produced in China is 0, the tensile strength decreases by 0.05 MPa, 0.16 MPa, and 0.20 MPa, and the decrease is small.

All in all, when the tensile strength of HFRCC is the first objective, the tensile strength of S0.4C0J2 is the highest, that is, the volume content of steel fiber is 0.4%, the volume content of domestic PVA fiber is 0, and the volume content of daily PVA fiber is 2%. This mix ratio is poor in economy. Considering the economic and practical engineering needs, S0.2C1.5J0.5 and S0.4C2.0J0 are the optimal mix ratio, that is, when the volume content of steel fiber is 0.2% and 0.4%, the replacement rate of domestic PVA fiber is 75% and 100%, respectively. Compared with S0.4C0J2, the reduction in tensile strength is about 5%; the reduction in tensile strength is not obvious, and the value for money is the best.

#### 3.2.2. Effect of the Replacement Rate of PVA Fibers Produced in China on the Compressive Strength of HFRCC

At the initial stage of loading the test block, the stress force is small and has no obvious change characteristics. However, with the increase in load, when the load reaches 30–40% of the ultimate strength of the test block, vertical fine cracks begin to appear in the test block, and these cracks gradually expand and lengthen and new cracks appear. When the load reached about 80% of the ultimate load, the specimen not only showed horizontal deformation, but also vertical and oblique cracks appeared, and the sound of slight fibers pulling out and breaking could be heard. Finally, when the specimen reaches the ultimate load, the crack penetrates and causes the specimen to fail, and the specimen no longer bears the load.

**Figure 12:**
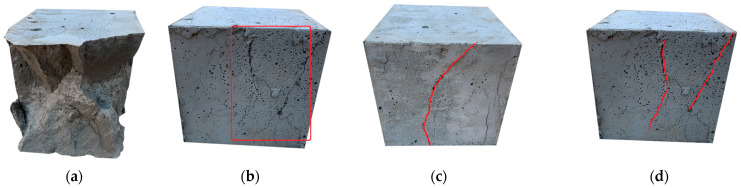
Axial compression failure mode: (**a**) Cone brittle failure; (**b**) slight bulge; (**c**) oblique crack; (**d**) vertical cracks and oblique cracks.

Under the condition of compression load, the brittle failure of ordinary concrete usually appears on the quadrangle cone, but the failure mode of HFRCC is obviously different from that of ordinary concrete. This is because the fiber plays a bridging role at the crack, which makes the transverse expansion of the middle part of the test block smaller, thus avoiding the external swelling phenomenon of the fiber concrete in the middle part [76,77]. At the same time, the bridge action can improve the compressive strength of the test block to a certain extent, and improve the overall performance of the material. Different from the brittle failure of ordinary concrete, which quickly breaks after it reaches the peak compressive load, HFRCC shows good compressive toughness. In the final failure stage of the specimen, the sound of fiber breaking and pulling out can be obviously heard. The failure of the specimen does not show brittle failure, and the surface of the specimen only shows diagonal cracks or slight bulking after pressure action. There is no corner slag or caving phenomenon; the whole is good. This indicates that HFRCC has a good toughness. Figure 12 shows some typical pictures of the test.

**Figure 13:**
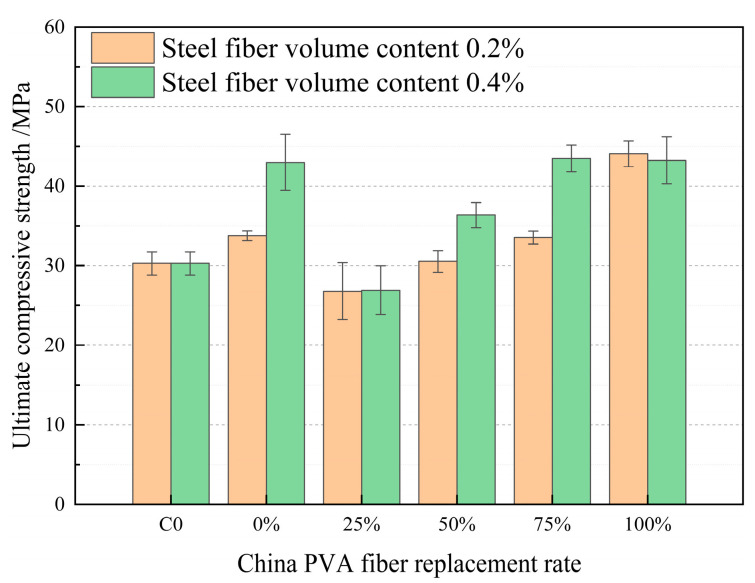
Comparison of compressive strength of PVA fibers produced in China at different volume addition rates.

According to the analysis of Figure 13, when the volume content of steel fiber is 0.2% and 0.4%, and the PVA fiber produced in China is used to replace the PVA fiber produced in Japan in HFRCC, the compressive strength of HFRCC presents a large fluctuation, and the compressive strength of HFRCC presents a “positive effect” and “negative effect” compared with the compressive strength of the matrix. Steel fiber and PVA fiber (produced in China and Japan) have different forms and mechanical properties. Steel fiber can effectively resist external loads and improve the compressive strength of concrete. PVA fiber is easy to deform and can absorb energy when stressed, which helps to resist the expansion of cracks, but its ability to improve compressive strength is relatively limited. When steel fiber is mixed into HFRCC, along with PVA fiber produced in China and PVA fiber produced in Japan, different characteristics of fiber may affect each other, resulting in an irregular improvement in compressive strength. At the same time, the degree of dispersion of the fiber will affect the effect of the fiber in the HFRCC. When the volume of steel fiber is the same, the compressive strength of HFRCC is “sensitive” to the change in the replacement rate of PVA fiber produced in China. When the volume content of steel fiber is 0.2% and the replacement rate of PVA fiber produced in China is 100%, the peak compressive strength is 44.07 MPa, which is significantly increased by 45.54% compared with the matrix compressive strength. When the volume content of steel fiber is 0.4% and the replacement rate of PVA fiber produced in China is 75% and 100%, the compressive strength is 43.48 MPa and 43.24 MPa, respectively, which significantly increases compared with the compressive strength of the matrix; the increase rate is 43.59% and 42.8% respectively, and the change rate of the compressive strength of both is 0.79%. The maximum difference in compressive strength of the mix ratio of the above three groups is 0.83 MPa, the maximum reduction is 2.74%, and the change range is small.

All in all, S0.2C2.0J0 has the highest compressive strength, that is, the volume content of steel fiber is 0.2%, the replacement rate of PVA fiber produced in China is 100%, and the compressive strength and economy are the best. The compressive strength of S0.4C1.5J0.5 and S0.4C2.0J0 is not much different from that of S0.2C2.0J0, that is, when the volume content of steel fiber is 0.2% and 0.4%, the replacement rate of PVA fiber produced in China is 75% and 100%.

#### 3.2.3. Effect of the Replacement Rate of PVA Fiber Produced in China at Different Volume Contents on the Bending Strength of HFRCC

**Figure 14:**
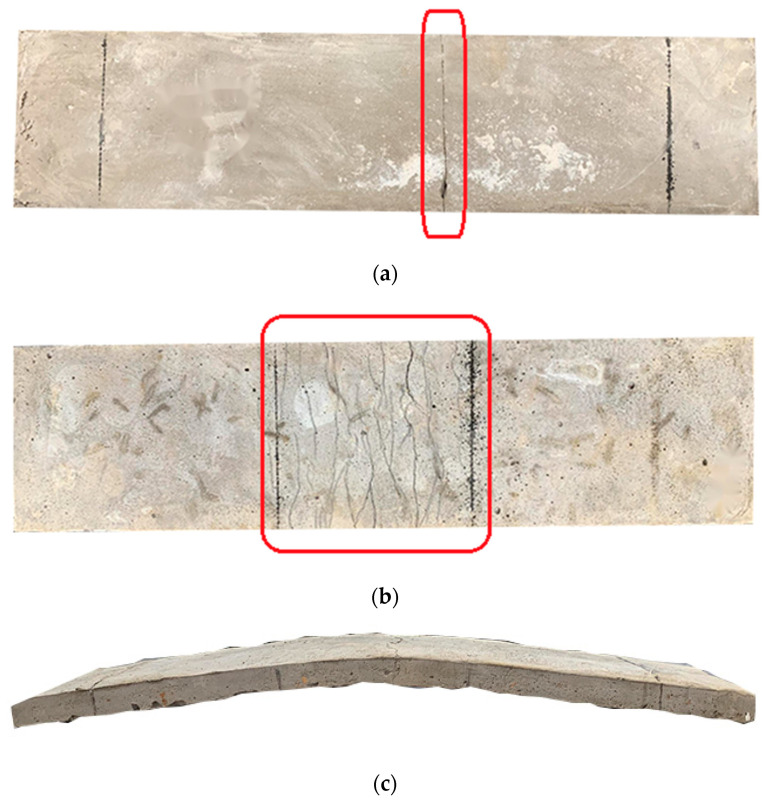
Four-point bending failure pattern: (**a**) C0 blank group brittle failure; (**b**) ductile destruction front; (**c**) ductile failure side.

By observing the loading process of thin plate specimens, the following changes and phenomena can be observed. In the initial loading process, the specimen was in the elastic stage, the flexural load–deformation curve showed a linear increase, the microcrack did not expand in the HFRCC, and no cracks were observed on the specimen surface. With the increase in load, multiple microcracks appeared when the HFRCC specimen reached the initial cracking stress, usually appearing at two loading points or midspan of the pure bending section, and the bearing capacity of the specimen began to decline. In this process, steel fiber and PVA fiber play an important role in bridging, delaying crack propagation [68,69]. As the number of cracks increased gradually, the flexural load–deflection curve of HFRCC began to fluctuate. Eventually, the specimen gradually lost its load-bearing capacity, exhibited bending characteristics, and with the sound of fiber breaking, a small number of concrete fragments fell out of the cracks. When the peak bending load is reached, the crack continues to expand until the fiber breaks and leads to failure. HFRCC presented a series of characteristics under load, including microcracks, bending properties, fiber bridging, and crack increase [70,71]. The testing machine stopped loading, and recorded and completed the mapping of the full curve of bending load and deflection. Different from the uniaxial tensile test, the specimen has a large bending deformation but no fracture after unloading the residual load, and it still has a certain plastic recovery ability. The bending failure morphology of some specimens is shown in Figure 14, where the red box is the crack location.

**Figure 15:**
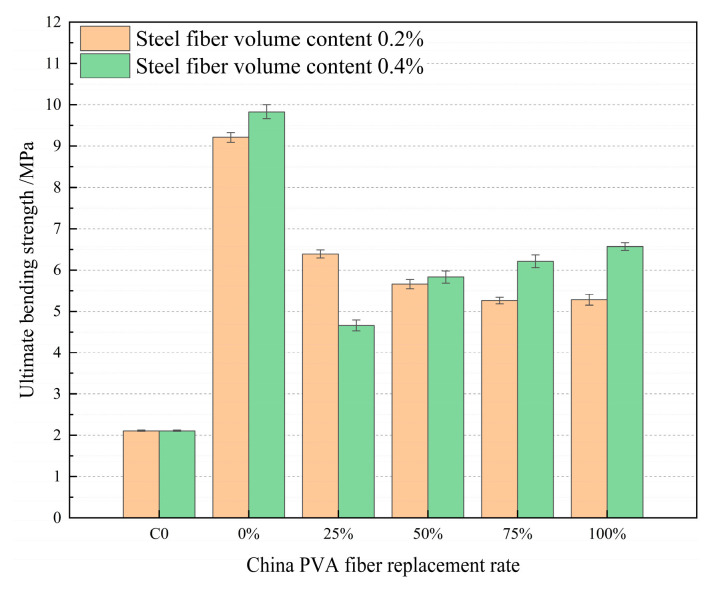
Comparison of flexural strength of PVA fibers produced in China at different volume addition rates.

According to the analysis of Figure 15, when the volume content of steel fiber is 0.2% and 0.4%, PVA fiber produced in China is used to replace PVA fiber produced in Japan in HFRCC, and the bending strength of HFRCC is greatly improved compared with the matrix. This is because when steel fiber and PVA fiber interact with the matrix, the bending strength of HFRCC is greatly improved. Steel fibers provide high-strength bridging during bending, while PVA fibers improve the overall toughness and crack resistance of the material [78,79]. Different fibers work at different scales and stages; PVA fibers limit the expansion of small cracks, steel fibers limit larger cracks, and this synergistic effect makes HFRCC have better deformation ability and a higher crack resistance when bending. When the volume content of steel fiber is 0.2% and the replacement rate of PVA fiber produced in China is 0, that is, the volume content of PVA fiber produced in Japan is 2%, the peak bending strength is 9.21 MPa, which significantly increases compared with the bending strength of the matrix by 336.49%. When the volume content of steel fiber is 0.4% and the replacement rate of PVA fiber produced in China is 0, that is, the volume content of PVA fiber produced in Japan is 2%, the peak bending strength is 9.83 MPa, which is significantly increased compared with the bending strength of the matrix, and the increase rate is 365.88%. When the volume content of steel fiber is 0.2%, the replacement rate of PVA fiber produced in China is 25%, and the bending strength of HFRCC is 6.39 MPa, which increases 202.84% compared with the bending strength of the matrix. When the volume content of steel fiber is 0.4%, the replacement rate of PVA fiber produced in China is 100%, and the bending strength of HFRCC is 6.57 MPa, which increases 211.37% compared with the bending strength of the matrix. The maximum difference in bending strength of the mix ratio of the above four groups is 3.44 MPa.

All in all, when the bending strength of HFRCC is the first objective, the bending strength of S0.2C0J2 is the highest, that is, the volume content of steel fiber is 0.2%, the volume content of PVA fiber produced in China is 0, and the volume content of PVA fiber produced in Japan is 2%. This mix ratio is poor in economic terms. If a comprehensive consideration is taken, S0.2C0.5J1.5 and S0.4C2.0J0 are the optimal mix ratios, that is, when the volume content of steel fiber is 0.2% and 0.4%, respectively, the replacement rate of PVA fiber produced in China is 25% and 100% respectively, and the actual demand of the project is met, so the value for money is the best.

## 4. Multi-Objective Optimization of Principal Component Analysis

It is not practical to maximize the tensile strength, compressive strength, and bending strength of HFRCC by a specific mix ratio, and it is even difficult to infer a reasonable range of the mix ratio design variables. The principal component analysis method is used to study the balance relationship between influencing factors and indicators from the test data, and the correlation analysis of variables is combined to find the indicators that should be paid attention to in the optimization of HFRCC performance, so as to predict and optimize an optimal mix ratio design scheme.

### 4.1. Principle of Principal Component Analysis

Principal component analysis (PCA) is the transformation of different variables using multivariate statistical analysis as the medium, and finally transforming multiple variables into several uncorrelated comprehensive indicator variables, so as to achieve the dimensionality reduction of variables and simplify related research problems. The main calculation theory is as follows [80,81,82]:

(1) There are *n* evaluation objects, and each evaluation object is described by *p* evaluation indicators. The evaluation index values $x_{1},\;x_{2},x_{3}\ldots ,x_{n}$, form a raw data matrix $x={\left(x_{ij}\right)}_{mn}$ of the order m×n, as shown in Equation (1):

(1) $$X=\left[\begin{array}{c} x_{1}^{T}\\ x_{2}^{T}\\ \vdots \\ x_{n}^{T} \end{array}\right]=\left[\begin{array}{ccc} \begin{array}{cc} x_{11} & x_{12}\\ x_{21} & x_{22} \end{array} & \cdots & \begin{array}{c} x_{1p}\\ x_{2p} \end{array}\\ \vdots & \ddots & \vdots \\ \begin{array}{cc} x_{n1} & x_{n2} \end{array} & \cdots & x_{np} \end{array}\right]\;\;\;$$

(2) The *p* vectors $X_{1},X_{2},\cdots X_{n}$ of the original matrix *X* are linearly transformed and the mathematical model is established, as shown in Equation (2):

(2) $$\left\{\begin{array}{c} maxvar\left(F_{r}\right)=var\left(\sum_{r=1}^{n}a_{r}x_{r}\right)=A^{T}VA\\ s.t.A^{T}A=\sum_{r=1}^{n}a_{r}^{2}=1 \end{array}\right.$$

Type: $A={\left[a_{1},a_{2,}\cdots a_{n}\right]}^{T}$, $a_{1},a_{2,}\cdots a_{n}$ for *n* linear combination of the original matrix coefficient, $var\left(F_{r}\right)$ is the variance of $F_{r}$, $F_{r}$ represents the magnitude of the difference of the combined side of $X_{1},X_{2},\cdots X_{n}$. The difference of the combined side is the largest when r=1, and the difference of the combined side is the smallest when r=n, as shown in Equation (3):

(3) $$var\left(F_{1}\right)\geq var\left(F_{2}\right)\geq \cdots \geq var\left(F_{n}\right)$$

(3) Establish the characteristic equation about R, obtain the eigenvalue $\lambda_{1}\geq \lambda_{2}\geq \cdots \geq \lambda_{p}\geq 0$ by Jacobi solution, and obtain the corresponding eigenvector $\left\|e_{i}\right\|$ according to the eigenvalue $\lambda_{i}$. The weighted arithmetic average is applied for synthesis, and the contribution rate of variance of each component is taken as the weight, as shown in Equations (4) and (5):

(4) $$\left|R-\lambda E\right|=0\;\;\;\;\;\;\;\;\;\;\;\;\;$$

(5) $$f=\frac{\lambda_{1}F_{1}+\lambda_{2}F_{2}+\cdots +\lambda_{k}F_{k}}{\sum_{i=1}^{p}\lambda_{i}}$$

Type: $\lambda_{1},\;\lambda_{2},\;\cdots ,\;\lambda_{k}$ is the eigenvalue of the covariance matrix of the matrix *X*

(4) Calculate the variance contribution rate $\alpha_{k}$ and cumulative variance contribution rate $\beta_{k}$ of principal components to determine the number of principal components. According to the cumulative variance contribution rate $\beta_{k}\geq 85\%$, the number of principal components is determined, and the variance contribution rate $\alpha_{k}$ of principal components is used to explain the corresponding index of each principal component, so as to achieve the purpose of transforming multiple variables into several unrelated comprehensive index variables, as shown in Equations (6) and (7):

(6) $$\alpha_{k}=\frac{\lambda_{k}}{\sum_{i=1}^{n}\lambda_{i}}$$

(7) $$\beta_{k}=\frac{\sum_{i=1}^{k}\lambda_{i}}{\sum_{i=1}^{n}\lambda_{i}}\geq 85\%\;\;\;\;$$

(5) The principal component matrix is used to complete the comprehensive analysis and evaluation of the target problem, and the optimal mix ratio of HFRCC with multiple objectives is finally obtained, as shown in Equation (8):

(8) $$Z=\sum_{I=1}^{P}\alpha_{k}e_{i}=\alpha_{1}e_{1}+\alpha_{2}e_{2}+\cdots +\alpha_{p}e_{p}$$

### 4.2. HFRCC Multi-Objective Principal Component and Index Analysis Calculation

This experiment focuses on the influence of steel fiber volume content, PVA fiber volume content produced in China, and PVA fiber volume content produced in Japan on the tensile strength, compressive strength, and bending strength of HFRCC. The multi-objective principal component and index analysis of HFRCC select the test data of the multi-factor optimization method. The test sample model of HFRCC was established according to Equation (1), as shown in Table 8.

#### 4.2.1. Correlation Analysis of HFRCC Influencing Factors

**Figure 16:**
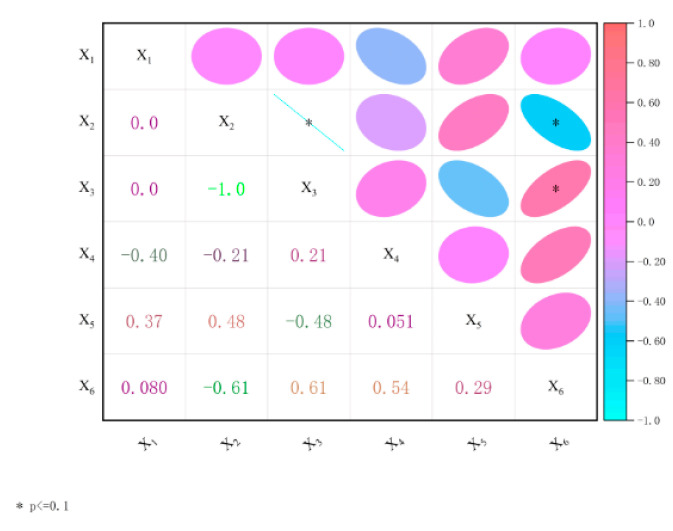
Correlation analysis of properties of HFRCC.

In order to quantify the degree of correlation between the volume content of steel fiber, the volume content of PVA fiber produced in China and the volume content of PVA fiber produced in Japan and the tensile strength, compressive strength, and bending strength of HFRCC, the Correlation Plot App in the Origin Pro 2022 software was applied to perform correlation analysis calculations. Figure 16 shows the HFRCC performance correlation analysis results. The numbers in the lower left part are the *R*-value of the correlation coefficient after linear fitting, the color depth of the ellipse in the upper right part corresponds to the *R*-value, and the asterisk * in the ellipse represents the significant correlation of the fitting parameters, where p≤0.1.

The analysis of Figure 16 shows that there is a significant negative correlation between the volume content of PVA fiber produced in China and that of PVA fiber produced in Japan, *R* = −1.0. The volume content of PVA fiber produced in China has a significant negative correlation with the bending strength (*R* = −0.61). There is a significant positive correlation between the volume content of PVA fiber produced in Japan and the bending strength (*R* = 0.61). In summary, the bending strength is significantly correlated with the volume content of PVA fiber produced in China and PVA fiber produced in Japan, respectively, and presents an opposite effect, with the same correlation coefficient *R* value.

#### 4.2.2. HFRCC Performance Model Variable Commonality

Calculate the sum of squares of the loads of the six variables on all common factors, the common degree. The degree of commonality represents the degree to which the six variables can be explained by the extracted principal components. The calculation results of the commonality of each variable are shown in Table 9. According to the analysis of Table 9, the common degree of the extracted variables is greater than 85%, indicating that the extracted common factors can represent more than 85% of the information in the original variables, and the factor analysis effect is good, and all variables can be explained.

#### 4.2.3. Analysis of Principal Component Eigenvalue and Variance Contribution Rate of HFRCC Performance Model

**Figure 17:**
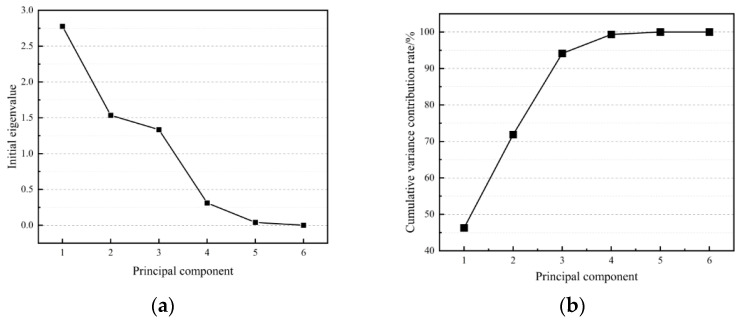
Interpretive ratio graph of HFRCC variance: (**a**) Interpretive ratio diagram of variances of each principal component; (**b**) interpretive ratio diagram of variances accumulative of principal components.

**Figure 18:**
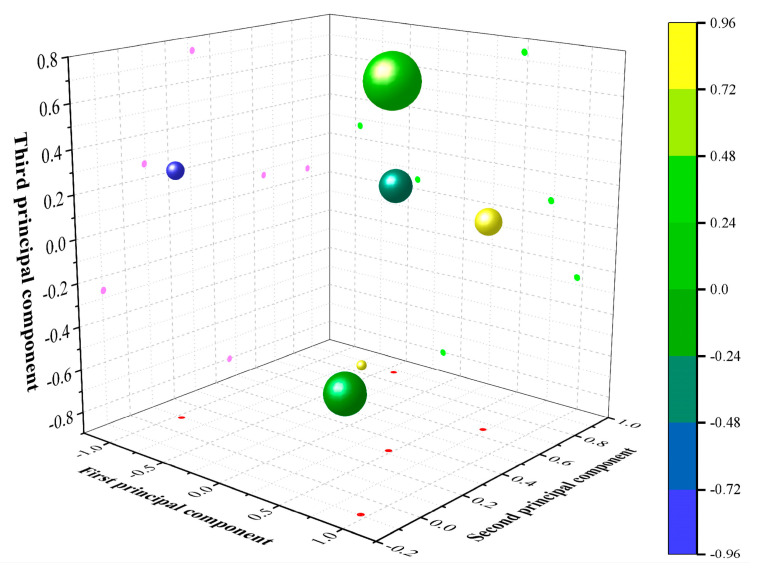
Spatial distribution of principal component index of HFRCC.

The principal component eigenvalues and variance contribution rates of the HFRCC performance model were calculated by Equations (6) and (7), and the results are shown in Table 10. The interpretation ratio of the variance of each component is also called the “gravel chart”, and it is shown in Figure 17. As can be seen from Table 10, the interpretation ratios of variance of each principal component are 2.777, 1.536, 1.333, 0.313, 0.040, and −2.220 × 10^−16^, respectively. By observing Figure 17a, it can be seen that the variance interpretation ratio of *F*_1_, *F*_2_, and *F*_3_ is all greater than 1, and the variance interpretation ratio of *F*_3_ to *F*_4_ suddenly drops by 76.63%, which is a large margin. Therefore, most of the variance is explained through the first three principal components, namely *F*_1_, *F*_2_, and *F*_3_. In this paper, a cumulative contribution rate of over 85% was adopted to analyze the performance model. The interpretation ratio of the cumulative variance of each principal component is shown in Figure 17b. The cumulative contribution rate of *F*_1_, *F*_2_, and *F*_3_ has reached 94.11%, which is a relatively high level. Figure 18 is the spatial distribution diagram of indicators in *F*_1_, *F*_2_, and *F*_3_, from which the distribution of indicators in the principal components can be seen, and the importance and correlation of indicators in different principal components can also be visually seen.

To sum up, the cumulative variance contribution rate of *F*_1_, *F*_2_, and *F*_3_ is greater than 85% and the variance interpretation ratio of each principal component is greater than 1, indicating that it is scientific and reasonable that *F*_1_, *F*_2_, and *F*_3_ can replace the six original variables. The dimensionality of the six original variables is reduced into three principal components to achieve the purpose of dimensionality reduction and give *F*_1_, *F*_2_, and *F*_3_ new comprehensive significance.

**Figure 19:**
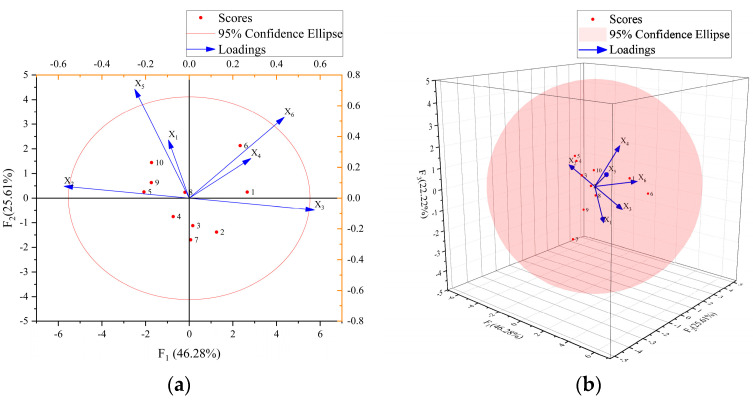
HFRCC load and score biplot: (**a**) Two-dimensional model; (**b**) three-dimensional model.

Table 11 shows the variance contribution rate of the first three principal components and the load matrix of the six original variables, which is the projected length of the six original variables on the coordinate axis in Figure 19. Figure 19a shows the correlation and load values of *F*_1_ and *F*_2_. Figure 19b shows the correlation and load values of *F*_1_, *F*_2_, and *F*_3_.

As can be seen from Table 11, the first principal component *F*_1_ is 46.277%, which is mainly determined by the volume content of PVA fiber produced in China, the volume content of PVA fiber produced in Japan, and the bending strength, and the factor load is −0.956, 0.956, and 0.726, respectively. The volume content of PVA fiber produced in China and PVA fiber produced in Japan is significantly correlated and negatively correlated, accounting for 51.87% of *F*_1_, which is consistent with the analysis results in 3.2.3. There is a negative correlation between the volume content of PVA fiber produced in China and the bending strength. The increase in the volume content of PVA fiber produced in China will reduce the bending strength of HFRCC, accounting for 38.74% of *F*_1_. There is a positive correlation between the volume content of PVA fiber produced in Japan and the bending strength. The increase in the volume content of PVA fiber produced in Japan will enhance the bending strength of HFRCC, accounting for 38.74% of *F*_1_. The volume content, tensile strength, and compressive strength of steel fiber have little influence on the first principal component *F*_1_.

The second principal component *F*_2_ is 25.605%, which is mainly determined by the volume content of steel fiber, compressive strength, and bending strength, and the factor loads are 0.468, 0.879, and 0.651, respectively. There is a positive correlation between the volume content of steel fiber and the compressive strength. The increase in the volume content of steel fiber enhances the compressive strength of HFRCC, accounting for 53.67% of F2. There is a positive correlation between the volume content of steel fiber and the flexural strength. The increase in the volume content of steel fiber enhances the flexural strength of HFRCC, accounting for 44.58% of *F*_2_. All in all, the increase in the volume content of HFRCC steel fiber can promote compressive strength and bending strength, and the enhancement effect of the compressive strength is better. The volume content of PVA fiber produced in China, the volume content of PVA fiber produced in Japan, and the tensile strength have little influence on the second principal component *F*_2_.

The third principal component *F*_3_ is 22.223%, which is mainly determined by the volume content of steel fiber and tensile strength, and the factor loads are −0.802 and 0.737, respectively. There is a negative correlation between the volume content of steel fiber and the tensile strength. The increase in the volume content of steel fiber will reduce the tensile strength of HFRCC, accounting for 70.31% of *F*_3_. PVA fiber volume content produced in China, PVA fiber volume content produced in Japan, compressive strength, and bending strength have little influence on the third principal component *F*_3_.

In summary, increasing the volume content of steel fiber can promote the growth of compressive strength and bending strength, and inhibit the growth of tensile strength. The volume content of PVA fiber produced in China will inhibit the growth of bending strength, while the volume content of PVA fiber produced in Japan will promote the growth of bending strength.

### 4.3. Calculation and Analysis of Comprehensive Performance Index of HFRCC

The score coefficient matrix of each component of the HFRCC performance model is shown in Table 12. The weight of the six variables in principal components *F*_1_, *F*_2_, and *F*_3_ can be obtained from Equation (8), and the relationship between principal components and variables can be obtained:

$$F_{1}=-0.057X_{1}-0.344X_{2}+0.344X_{3}+0.170X_{4}-0.150X_{5}+0.261X_{6}$$

$$F_{2}=0.305X_{1}+0.062X_{2}-0.062X_{3}+0.208X_{4}+0.572X_{5}+424X_{6}$$

$$F_{2}=-0.601X_{1}+0.199X_{2}-0.199X_{3}+0.553X_{4}+0.036X_{5}+0.054X_{6}$$

The comprehensive performance indicators of HFRCC variables can be obtained from the following formula, and the results are shown in Table 13:

As can be seen from Table 13 and Table 14, S0.4C0J2 has the highest comprehensive score, and the volume content of PVA fiber produced in Japan is 2%, which can be considered as the optimal mix ratio, but the value for money is poor and the cost is high. The comprehensive score of S0.4C2.0J0 ranked second. The volume content of PVA fiber produced in China was 2%, and compared with S0.4C0J2, the tensile strength decreased by 0.21 MPa, a decrease of 5.45%. Compressive strength increased by 0.28 MPa, or 0.62%. The bending strength was reduced by 3.84 MPa (33.19%). The preparation cost of HFRCC per cubic meter was reduced by 9962 yuan, a decrease of 88.25%.

In summary, the mix ratio of HFRCC was optimized according to the comprehensive evaluation results of the performance model. The steel fiber content was 0.2–0.4%, and the PVA fiber produced in China was mixed with the volume content of PVA fiber produced in Japan with the replacement rate of 50%, 75%, and 100%. Its tensile strength is greater than 2.5 MPa, its compressive strength is greater than 30 MPa, and its bending strength is greater than 6 MPa. Combined with a variable correlation analysis, we focus on the indicators with a significant correlation when optimizing HFRCC mix ratio performance, and combine the degree of importance to ensure performance and reduce the HFRCC preparation cost, which has certain reference significance for practical engineering.

## 5. Conclusions

The factor optimization method was used to investigate the three performance indexes of HFRCC, tensile strength, compressive strength, and bending strength, and a corresponding mix ratio for ultimate strength was obtained, that is, the tensile strength of S0.4C0J2 is 3.66 MPa, and the compressive strength of S0.2C2.0J0 is 44.07 MPa. S0.4C0J2’s bending strength is 9.83 MPa. Compared with the matrix performance index, the maximum increase in tensile strength is 155.94%, the maximum increase in compressive strength is 45.54%, and the maximum increase in bending strength is 365.88%.

Principal component analysis (PCA) was applied to transform the different dimensions of HFRCC steel fiber volume content, PVA fiber volume content produced in China, PVA fiber volume content produced in Japan, tensile strength, compressive strength, and bending strength among the six variables into dimensionless indexes to achieve the purpose of dimensionality reduction. The mix ratio and mechanical properties of HFRCC were evaluated comprehensively.

The weight proportion of each factor affecting HFRCC performance is as follows: The volume content of PVA fiber produced in China, the volume content of PVA fiber produced in Japan, and the bending strength accounted for 46.28%. The volume content, compressive strength, and bending strength of steel fiber accounted for 25.61%. The volume content and tensile strength of steel fiber accounted for 22.22%. Combined with a variable correlation analysis, these indicators should be focused on when conducting a performance optimization based on HFRCC.

A comprehensive factor optimization method and principal component analysis (PCA) were used to analyze the comprehensive performance indicators of HFRCC, achieve the preset performance and economic targets, and obtain the range of steel fiber content and the replacement rate of PVA fiber produced in China to PVA fiber produced in Japan that is most suitable for engineering applications. That is, the steel fiber content is 0.2–0.4%, the PVA fiber produced in China is blended with the volume content of the PVA fiber produced in Japan with the replacement rate of 50%, 75%, and 100%, and the tensile strength is greater than 2.5 MPa, the compressive strength is greater than 30 MPa, the bending strength is greater than 6 MPa, and the maximum cost reduction is 88.25%.

## Figures and Tables

**Table 1:** Key physical and mechanical properties of Portland cement.

Initial Setting Time/min	Final Setting Time/min	Stability	Compressive Strength/MPa		Flexural Strength/MPa	
			3d	28d	3d	28d
135	168	Up to standard	22.7	46.7	4.8	8.4

**Table 2:** Chemical composition of Portland cement.

Ingredient	SiO_2_	Al_2_O_3_	Fe_2_O_3_	MgO	CaO	R_2_O	SO_3_	Firing Vector
Content /%	14.52	1.66	0.15	0.17	31.94	0.22	45.38	0.25

**Table 3:** Physical properties of fly ash.

Fineness (45 μm Square Hole Sieve Residue)/%	Water Requirement Ratio/%	Water Content/%	Alkali Content/%	Activity Index/%
8.5	94	0.5	1.3	76

**Table 4:** Chemical composition of fly ash.

Ingredient	SiO_2_	Al_2_O_3_	Fe_2_O_3_	SO_3_	Firing Vector
Content/%	75			1.1	3.8

**Table 5:** Related performance parameters of steel fiber and PVA fiber.

Fiber Type	Length/mm	Diameter/μm	Tensile Strength/MPa	Dry Fracture Elongation/%	Density/(g/cm^3^)	Young’s Modulus/GPa
Steel fiber	13	200	2850	3.20	7.80	210
Japan PVA fiber	12	39	1620	7	1.30	42.80
China PVA fiber	12	15.09	1830	6.90	1.29	40

**Table 6:** Single factor test mix ratio design and test results of HFRCC.

Test Number	Fiber Volume Content %		Experimental Result/MPa		
	China PVA Fiber	Japan PVA Fiber	Tensile Strength	Compressive Strength	Bending Strength
C0	0	0	1.43	30.28	2.11
C0.4J0	0.4	0	2.62	29.14	3.43
C0.8J0	0.8	0	2.28	26.86	3.59
C1.2J0	1.2	0	2.30	30.69	4.71
C1.6J0	1.6	0	3.08	32.04	4.12
C2.0J0	2.0	0	3.68	34.39	4.35
C2.2J0	2.2	0	3.31	28.21	4.38
C0J0.4	0	0.4	2.89	29.00	2.02
C0J0.8	0	0.8	2.16	26.10	3.01
C0J1.2	0	1.2	2.38	30.52	4.38
C0J1.6	0	1.6	3.31	31.23	5.13
C0J2.0	0	2.0	4.23	34.09	5.23
C0J2.2	0	2.2	3.74	29.94	4.65

**Table 7:** Multi-factor test mix ratio design and test results of HFRCC.

Test Number	Fiber Volume Content %			Experimental Result/MPa		
	Steel Fiber	China PVA Fiber	Japan PVA Fiber	Tensile Strength	Compressive Strength	Bending Strength
C0	0	0	0	1.43	30.28	2.11
S0.2C0J2	0.2	0	2.0	3.61	33.77	9.21
S0.2C0.5J1.5	0.2	0.5	1.5	3.37	26.79	6.39
S0.2C1.0J1.0	0.2	1.0	1.0	3.37	30.53	5.66
S0.2C1.5J0.5	0.2	1.5	0.5	3.50	33.54	5.26
S0.2C2.0J0	0.2	2.0	0	3.21	44.07	5.28
S0.4C0J2	0.4	0	2.0	3.66	42.98	9.83
S0.4C0.5J1.5	0.4	0.5	1.5	2.57	26.92	4.66
S0.4C1J1	0.4	1.0	1.0	3.36	36.36	5.83
S0.4C1.5J0.5	0.4	1.5	0.5	2.48	43.48	6.21
S0.4C2.0J0	0.4	2.0	0	3.46	43.24	6.57

**Table 8:** Performance model parameters of HFRCC.

Test Number	Fiber Volume Content %			Experimental Result/MPa		
	Steel Fiber	China PVA Fiber	Japan PVA Fiber	Tensile Strength	Compressive Strength	Bending Strength
	*X*_1_	*X*_2_	*X*_3_	*X*_4_	*X*_5_	*X*_6_
S0.2C0J2	0.2	0	2.0	3.61	33.77	9.21
S0.2C0.5J1.5	0.2	0.5	1.5	3.37	26.79	6.39
S0.2C1.0J1.0	0.2	1.0	1.0	3.37	30.53	5.66
S0.2C1.5J0.5	0.2	1.5	0.5	3.50	33.54	5.26
S0.2C2.0J0	0.2	2.0	0	3.21	44.07	5.28
S0.4C0J2	0.4	0	2.0	3.66	42.98	9.83
S0.4C0.5J1.5	0.4	0.5	1.5	2.57	26.92	4.66
S0.4C1J1	0.4	1.0	1.0	3.36	36.36	5.83
S0.4C1.5J0.5	0.4	1.5	0.5	2.48	43.48	6.21
S0.4C2.0J0	0.4	2.0	0	3.46	43.24	6.57

**Table 9:** HFRCC performance model variable commonality.

Project	$X_{1}$	$X_{2}$	$X_{3}$	$X_{4}$	$X_{5}$	$X_{6}$
Start	1.000	1.000	1.000	1.000	1.000	1.000
Extraction commonality	0.888	0.994	0.994	0.868	0.948	0.956

**Table 10:** Principal component characteristic value and variance contribution rate of HFRCC performance model.

Principal Component	Initial Eigenvalue			Extract the Sum of Loads Squared		
	Total	Variance Contribution Rate/%	Cumulative Variance Contribution Rate/%	Total	Variance Contribution Rate/%	Cumulative Variance Contribution rate/%
*F_1_*	2.777	46.277	46.277	2.777	46.277	46.277
*F_2_*	1.536	25.605	71.882	1.536	25.605	71.882
*F_3_*	1.333	22.223	94.105	1.333	22.223	94.105
*F_4_*	0.313	5.222	99.327			
*F_5_*	0.040	0.673	100.000			
*F_6_*	2.220 × 10^−16^	3.701 × 10^−15^	100.000			

**Table 11:** Factor load matrix for performance model of HFRCC.

Project	Principal Component		
	*F*_1_ (46.277%)	*F*_2_ (25.605%)	*F*_3_ (22.223%)
$X_{1}-$Steel fiber volume ratio (volume fraction)	−0.159	0.468	−0.802
$X_{2}-$China PVA fiber volume ratio (volume fraction)	−0.956	0.096	0.265
$X_{3}-$Japan PVA fiber volume ratio (volume fraction)	0.956	−0.096	−0.265
$X_{4}-$Tensile strength	0.472	0.320	0.737
$X_{5}-$Compressive strength	−0.417	0.879	0.048
$X_{6}-$Bending strength	0.726	0.651	0.072

**Table 12:** Score coefficient matrix of each component in performance model of HFRCC.

Project	Principal Component		
	*F*_1_	*F*_2_	*F*_3_
$X_{1}-$Steel fiber volume ratio (volume fraction)	−0.057	0.305	−0.601
$X_{2}-$China PVA fiber volume ratio (volume fraction)	−0.344	0.062	0.199
$X_{3}-$Japan PVA fiber volume ratio (volume fraction)	0.344	−0.062	−0.199
$X_{4}-$Tensile strength	0.170	0.208	0.553
$X_{5}-$Compressive strength	−0.150	0.572	0.036
$X_{6}-$Bending strength	0.261	0.424	0.054

**Table 13:** Synthetic evaluation results of HFRCC performance model.

**Test Number**	**Fiber Volume Content %**			**Comprehensive Evaluation Score**	**Ranking**	**Fiber Cost/(yuan/m^3^)**
	**Steel Fiber**	**China PVA Fiber**	**Japan PVA Fiber**			
S0.2C0J2	0.2	0	2.0	6.60	3	1214
S0.2C0.5J1.5	0.2	0.5	1.5	5.11	9	3710
S0.2C1.0J1.0	0.2	1.0	1.0	5.24	8	6205
S0.2C1.5J0.5	0.2	1.5	0.5	5.43	7	8700
S0.2C2.0J0	0.2	2.0	0	6.30	5	11,195
S0.4C0J2	0.4	0	2.0	7.62	1	11,289
S0.4C0.5J1.5	0.4	0.5	1.5	4.42	10	3818
S0.4C1J1	0.4	1.0	1.0	5.81	6	6308
S0.4C1.5J0.5	0.4	1.5	0.5	6.31	4	8798
S0.4C2.0J0	0.4	2.0	0	6.66	2	1327

Note: “Fiber cost” in the table is the price of the fiber required to configure 1 m^3^ of composite material, the currency type is CNY, the same as Table 14.

**Table 14:** Multifactor fiber content test fiber cost comparison.

Test Number	Fiber Volume Content %			Experimental Result/MPa			Fiber Cost/(yuan/m^3^)
	Steel Fiber	China PVA Fiber	Japan PVA Fiber	Tensile Strength	Compressive Strength	Bending Strength	
S0.4C0J2	0.4	0	2.0	3.85	45.24	11.57	11,289
S0.4C2.0J0	0.4	2.0	0	3.64	45.52	7.73	1327
				(0.21)	(−0.28)	(3.84)	(9962)

Note: In parentheses is the difference between S0.4C0J2 and S0.4C2.0J0.

## Data Availability

The original contributions presented in the study are included in the article, further inquiries can be directed to the corresponding author.
